# Dynamic primary resources, not just wild prey availability, underpin lion depredation of livestock in a savanna ecosystem

**DOI:** 10.1002/ece3.70208

**Published:** 2024-09-08

**Authors:** Kirby L. Mills, Emily Bennitt, Kai Zhu, Hattie L. A. Bartlam‐Brooks, Tatjana Y. Hubel, Alan M. Wilson, Neil H. Carter, Nathan J. Sanders

**Affiliations:** ^1^ Department of Ecology and Evolutionary Biology University of Michigan Ann Arbor Michigan USA; ^2^ Institute for Global Change Biology, University of Michigan Ann Arbor Michigan USA; ^3^ Okavango Research Institute, University of Botswana Maun Botswana; ^4^ School for Environment and Sustainability University of Michigan Ann Arbor Michigan USA; ^5^ Structure and Motion Laboratory Royal Veterinary College Hatfield UK

**Keywords:** global change, human–wildlife coexistence, NDVI, NDWI, pastoralism, predator–prey interactions, telemetry

## Abstract

Because it can lead to retaliatory killing, livestock depredation by large carnivores is among the foremost threats to carnivore conservation, and it severely impacts human well‐being worldwide. Ongoing climate change can amplify these human–wildlife conflicts, but such issues are largely unexplored, though are becoming increasingly recognized. Here, we assessed how the availability of primary resources and wild prey interact to shape large carnivore selection for livestock rather than wild prey (i.e., via prey switching or apparent competition). Specifically, we combined remotely sensed estimates of primary resources (i.e., water availability and primary productivity), wild prey movement, and 7 years (2015–2021) of reports for livestock depredation by African lions (*Panthera leo*) in the Makgadikgadi Pans ecosystem, Botswana. Although livestock depredation did not vary between wet versus dry seasons, analyses at finer temporal scales revealed higher incidences of livestock depredation when primary production, water availability, and wild prey availability were lower, though the effects of wild prey availability were mediated by water availability. Increased precipitation also amplified livestock depredation events despite having no influence on wild prey availability. Our results suggest that livestock depredation is influenced by the diverse responses of livestock, wild prey, and lions to primary resource availability, a driver that is largely overlooked or oversimplified in studies of human–carnivore conflict. Our findings provide insight into tailoring potential conflict mitigation strategies to fine‐scale changes in resource conditions to efficiently reduce conflict and support human livelihoods.

## Graphical abstract

Livestock depredation by African lions was driven by a combination of primary resources and wild prey availability. Depredation rates generally increased when resources were depleted, though the relationship between depredation and wild prey availability became decoupled when primary resources like water and forage quality were low. Conflict mitigation efforts should be tailored to fine‐scale temporal changes in resources, with consideration for both basal resources and species interactions that influence carnivore, wild prey, and livestock ecologies.

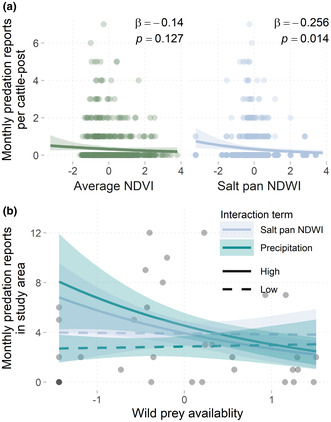

## 1 INTRODUCTION

Conflict between humans and large carnivores due to livestock depredation is a major threat to large carnivore conservation and human livelihoods (Braczkowski et al., 2023; Treves & Karanth, 2003). Human–carnivore conflicts are expected to intensify as climate change redistributes resources and wildlife in space and time (Abrahms et al., 2023; Fuller et al., 2016; Guiden et al., 2019; Tucker et al., 2018). Patterns in herbivore abundance and distribution can be interrupted by human‐caused environmental changes such as reduction in forage quality, intensified droughts, or the establishment of artificial waterpoints (Goswami et al., 2021; Harris et al., 2009; Middleton et al., 2013; Smit et al., 2007). Though fluctuations in wild prey availability are linked to livestock depredation rates (Amador‐Alcalá et al., 2013; Kabir et al., 2014; Valeix et al., 2012), the pathways through which bottom‐up changes in primary resources and wild prey availability may escalate livestock depredation and threaten vulnerable human livelihoods are largely unexplored.

Several studies have highlighted seasonal trends in livestock depredation by African lions (*Panthera leo*). Although the environmental drivers of lion depredation on livestock are not often explicitly tested, this seasonal variation is generally thought to be mediated by heterogeneity in the relationships among predators, wild prey, livestock, and primary resources. In some systems, livestock depredation by lions might increase in dry seasons, presumably when wild prey availability is limited, leading lions to switch from wild to domestic animals (McNutt et al., 2017; Schiess‐Meier et al., 2007). Alternatively, precipitation increases in some systems can lead lions to switch their prey selection to livestock, possibly because wild prey animals are dispersed more widely across a resource‐rich landscape and are thus less accessible (Olivier et al., 2022; Patterson et al., 2004; Sogbohossou et al., 2011). Apparent competition could also intensify livestock depredation when abundant wild prey support high densities of lions but become more inaccessible than livestock through effective predator‐avoidance strategies (Beattie et al., 2020; Hatton et al., 2015; Riginos, 2015). Understanding the ecological processes that underpin fine‐scale temporal variation in livestock depredation can enable more targeted and efficient conflict mitigation efforts (Miller & Schmitz, 2019; Wilkinson et al., 2020).

Here, we examine the relationships among resource availability, wild prey distributions, and livestock depredation by African lions (*Panthera leo*). Though they are an iconic species of conservation concern, lions are conflict‐prone throughout their range and cause severe economic impacts for local communities (Braczkowski et al., 2023; Di Minin et al., 2021). To examine how resources (specifically, water and vegetation that fundamentally support animal populations) shape temporal trends in wild prey availability and rates of livestock depredation by large carnivores, we combined remotely sensed estimates of dynamic water availability and vegetation greenness (a common proxy for primary productivity), hourly wild prey telemetry locations, and 7 years of incident reports for livestock depredation by African lions in northern Botswana. Specifically, we tested whether (1) higher water availability and vegetation greenness increase wild prey availability; (2) abundant wild prey directly reduces the incidence of livestock depredation by lions via prey switching or intensifies depredation via apparent competition; and (3) environmental resource availability versus wild prey availability is a stronger predictor of livestock depredation. Our results can inform management practices in complex social‐ecological systems and indicate that changes in climate‐driven resource availability may have unforeseen consequences for human–carnivore conflict in communities worldwide.

## 2 METHODS

### 2.1 Study area

**FIGURE 1:**
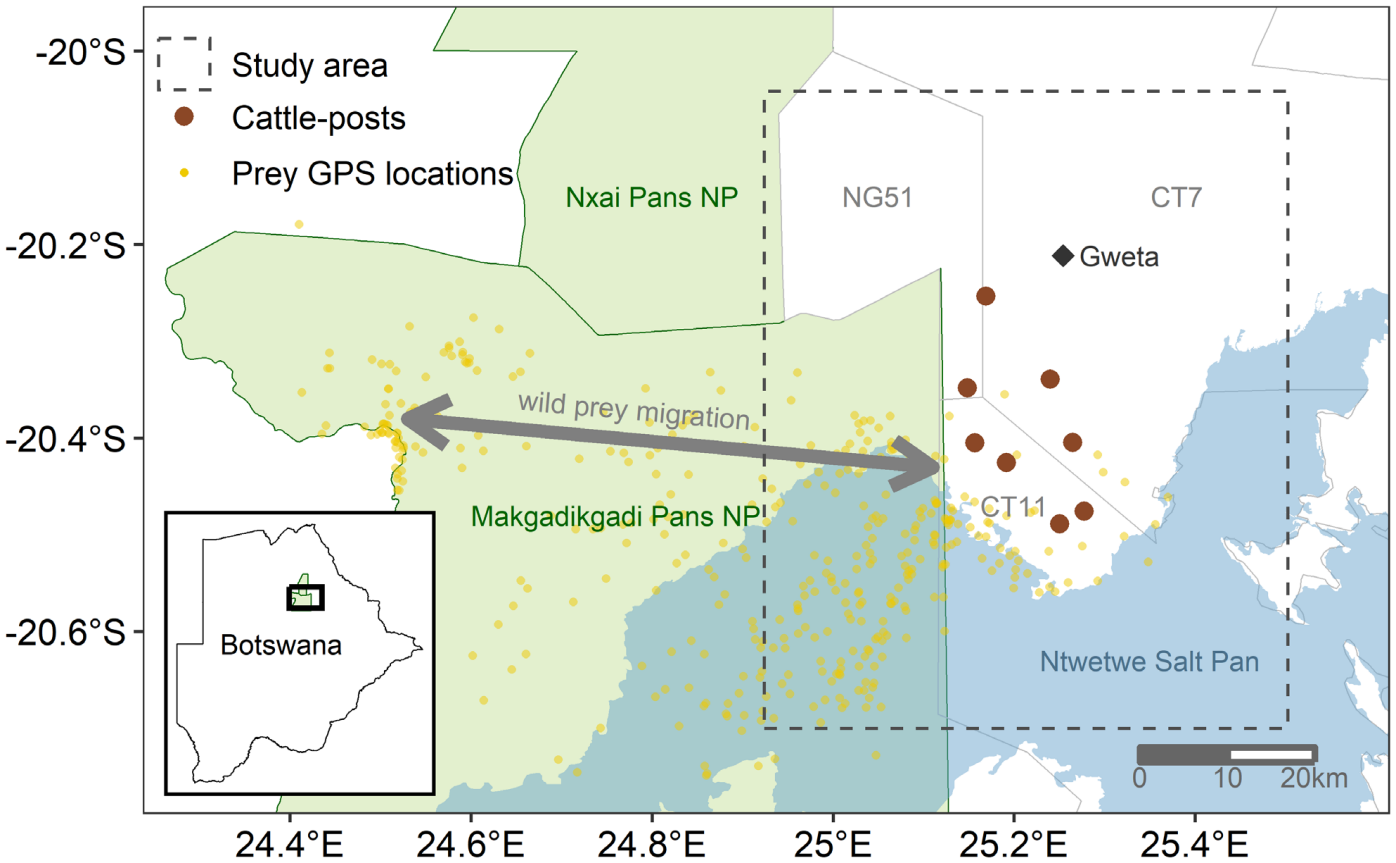
Study area location with respect to the Makgadikgadi and Nxai Pans National Parks (NPs) in northern Botswana. Larger, dark red points represent the locations of the eight cattle‐posts included in the study. Smaller, yellow points show the general distribution of prey GPS data during the study period using a random point for each collared individual per month. The gray arrow spanning the NPs depicts the general path of the seasonal migration of wild prey (i.e., zebra and wildebeest) from their eastern wet season range (~Dec–Apr) to their western dry season (~May–Nov) range. A map of the distribution of natural and artificial waterholes can be found in Bennitt et al. (2022), Biological Conservation.

The study area, referred to here as the Makgadikgadi Pans, covers approximately 5000 km^2^ in northern Botswana (24.9–25.5° E, 20.0–20.7° S) between the village of Gweta, the eastern boundary of the Makgadikgadi Pans National Park (MPNP), and the Ntwetwe salt pan (Figure 1). There are two broad categories of vegetation in the study area (Brooks, 2005): pan grassland closer to the salt pans (dominated by *Cenchrus ciliaris* and *Sporobolus ioclados* grasses) and mixed woodland elsewhere (primarily *Colophospermum mopane* and *Combretum imberbe* trees with *S. ioclados* grasses). The study area is composed of lands designated for photographic tourism (primarily CT11 in Figure 1) and livestock grazing (primarily in CT7 and NG51 in Figure 1). Livestock herds are housed at clusters of individual farms, locally referred to as cattle‐posts. Livestock are unpenned to graze unattended during the day, returning to the cattle‐posts in the evening for water and protection, though some animals (up to 13% of individuals) may not return to the cattle‐posts at night when they are most vulnerable to predators (Hemson et al., 2009).

The dominant wildlife in this system are plains zebra (*Equus quagga*) and blue wildebeest (*Connochaetes taurinus*), with populations numbering up to 24,000 and 10,000 individuals, respectively (Chase et al., 2015). Both species migrate en masse across the approximately 6000‐km^2^ MPNP between the wet and dry seasons. In the wet season (~December–April), zebra and wildebeest herds usually reside near the southeastern Ntwetwe salt pans where they forage and have access to mostly ephemeral water, though some artificial waterholes are also maintained. When water dwindles in the dry season (~May–November), the ungulates migrate to the Boteti River on the western MPNP border that generally provides potable water year‐round (Loveridge et al., 2010). However, changes in seasonal water availability, such as particularly wet years or year‐round pumping of artificial waterholes, can interrupt the timing of this seasonal migration. Notably, wildebeest populations are now more likely to remain near the salt pans during the dry season, probably in response to the installation of artificial waterholes in the area (Bennitt et al., 2022). The migration of wild prey has been suggested to impact rates of livestock depredation between seasons in this system, because lions preferentially prey on wildebeest and zebra but may increase selection for livestock prey when wild prey migrate away from lion home ranges (Hemson, 2003; Valeix et al., 2012). But given the recent changes in wildebeest migration patterns that may be linked to water availability, it is now unclear whether the availability of primary resources, wild prey, or a combination of both are the most important drivers of livestock depredation in the Makgadikgadi Pans system.

Other large ungulate species are present year‐round in the study area (such as greater kudu [*Tragelaphus strepsiceros*], gemsbok [*Oryx gazella*], and red hartebeest [*Alcelaphus buselaphus*]) that provide consistent prey resources for lions (Hayward & Kerley, 2005), but they occur at much lower densities relative to zebra and wildebeest (between 1400 and 2400 individuals reported by Valeix et al. (2012)) and are unlikely to exhibit similar fluctuations in abundance because of resource availability.

Most recently, lion density in eastern MPNP was estimated at 0.5 individuals per 100 km^2^ (Wildlife Conservation Research Unit, *unpublished data*). Unfortunately, this understudied system lacks up‐to‐date information on fine‐scale lion habitat selection. Lion sightings are most common within the national park boundaries and near the salt pan in CT11, but lions are known to leave the protected park and ecotourism areas to forage, sometimes resulting in livestock depredation. Though this evidence is anecdotal, it is supported by assessments of lion home ranges and foraging activity between 2001 and 2003 (Valeix et al., 2012). Although previous work suggests lions avoid areas near cattle‐posts if their primary migratory prey are present and move closer to cattle‐posts when migratory prey are absent (Valeix et al., 2012), it remains unknown how lion habitat selection responds to fluctuations in primary resources such as water. Lions are reported to be the most abundant large carnivore in the study area by local tourism operators and residents, though leopard (*Panthera pardus*), cheetah (*Acinonyx jubatus*), spotted hyena (*Crocuta crocuta*), and brown hyena (*Hyaena brunnea*) are also present (DEA, 2010; Ngaka, 2015). These sympatric predators generally exhibit relatively low dietary overlap with lions and do not show the same strong preference for wildebeest and zebra (Hayward & Kerley, 2008). Livestock predation by predators other than lions is thus less likely to fluctuate in response to pulses of migratory prey availability.

### 2.2 Livestock depredation

To measure rates of livestock depredation by lions in the study area, we used Problem Animal Control (PAC) data which are collected and maintained by Botswana's Department of Wildlife and National Parks (DWNP). PAC data are the basis for Botswana's national compensation program that reimburses residents for losses to wildlife to incentivize conservation efforts and reduce retaliatory killings of animals. Because residents receive no compensation for livestock losses to unprotected predators, such as spotted hyenas, or lower compensation rates for others, such as leopards and wild dogs, there is some potential for bias in the PAC data if livestock killings are falsely attributed to lions (LeFlore et al., 2019). However, PAC reports are generally verified by on‐the‐ground visits by DWNP officers, which should limit misclassifications of predators. Additionally, we do not expect this error to be associated with our explanatory variables of interest.

We received digitized PAC data from January 2015 to December 2021, courtesy of DWNP, wherein each report represents an occurrence of livestock depredation (including cattle, sheep, goats, donkeys, etc.) reported by cattle owners and verified by DWNP employees. Though the likelihood of residents reporting cases of livestock depredation could change over time due to socio‐economic trends or policy changes, we are unaware of any changes to the PAC program during the study period that could lead to inconsistency in reporting efforts. Because the PAC data include only the names of cattle‐posts and usually do not include the exact locations of livestock depredation events, we assigned the location of the reports to the geographic centroid of the cluster of farms making up each cattle‐post. Individual cattle‐post farms were identified and geolocated during fieldwork in the study area in June–July 2022. We filtered the PAC data to reports of livestock depredation (any livestock species) attributed to lions at cattle‐posts that are <20 km from MPNP and with >10 total PAC reports during the study period (>1.5 per month on average). In doing so, we restricted data to the eight cattle‐posts that were most likely to be influenced by patterns in wild prey and seasonal resource availability (Figure 1) while limiting analytical complications due to excessive zero‐inflation, as cattle‐posts farther from MPNP had far fewer reports of livestock depredation by lions because lions rarely reside in areas far from the park. We summed the total number of PAC reports for every month from January 2015 to December 2021 to create two datasets for subsequent modeling: (1) monthly PAC reports for all cattle‐posts together (i.e., total number of PAC reports per month, *N* = 84 months) and (2) monthly PAC reports for individual cattle‐posts (i.e., number of PAC reports per month × 8 cattle‐posts, *N* = 672).

### 2.3 Cattle‐post questionnaires

When identifying cattle‐post locations in June–July 2022, we gathered information on livestock herd sizes and perceptions of conflict from cattle‐post residents and workers using a brief questionnaire survey. At each cattle‐post where an owner, resident, or worker was available, we asked the following three questions: (1) Are livestock killings by predators becoming more or less frequent in the last 10 years?; (2) In the last 2–5 years, are more of your livestock killed by predators during the rainy or dry season?; and (3) Approximately how many livestock do you have (cattle, goats/sheep, other)? Questions about livestock killings were not specific to lions, and so respondents' answers are considered indicative of general trends in livestock depredation only. This research was considered exempt from institutional review by the University of Michigan IRB and was reviewed and approved by the University of Botswana IRB.

### 2.4 Wild prey availability

We used movement data for migratory wildebeest and zebra to represent pulses of wild prey availability in the study area for a subset of years in the study period. Between 2016 and 2018, 10 zebra and 18 wildebeest from independent social groups were fitted with GPS collars to track herd movements across MPNP (Bennitt et al., 2022). All collaring methods and data collection were conducted under research permits from the Ministry of Environment, Natural Resources Conservation and Tourism (EWT 3/3/8 XXXVIII, EWT 8/36/4 XXIV (199)) and were approved by the Ethics and Welfare Committee of the Royal Veterinary College (RVC 2013 1233). GPS collars were custom‐built by the Royal Veterinary College and designed to collect fixes every 5 min with pre‐programmed drop‐off mechanisms to be activated after 18 months of deployment (Curtin et al., 2018; Wilson et al., 2013).

We combined data for wildebeest and zebra to represent overall wild prey availability because both are known preferred prey for lions, and a difference in lion preferences between the two species has not been detected in this system (Hayward & Kerley, 2005; Valeix et al., 2012). We used the wild prey GPS locations, resampled to 1‐hour intervals, to assess relative wild prey availability per month by calculating the proportion of collared individuals' GPS locations that occur within the study area for each month between May 2016 and December 2018 (*N* = 32 months). We included only individuals with collar data available for at least 12 months of the study period (*N* = 17 individuals; 13 wildebeest and 4 zebra), and we excluded months for which an individual recorded fewer than 100 GPS locations. Our calculations resulted in a monthly measure of wild migratory prey availability that effectively reflects the relative intensity of use within the study area for all collared animals. Though our ability to detect patterns in overall wild prey availability may be limited by having GPS data for only a small proportion of the wildebeest and zebra populations and for only 3 years, we expect that the collared individuals are generally representative of the wild prey population distributions and responsiveness to resource availabilities (Bennitt et al., 2022).

### 2.5 Environmental data

We assessed three environmental variables to represent primary resource availability in the study system: (1) lagged precipitation, (2) surface water availability, and (3) primary productivity.

Monthly precipitation data were extracted from the CHIRPS terrestrial precipitation dataset (0.5‐degree resolution) averaged across the entire extent of the study area (Funk et al., 2014). We calculated the monthly lagged precipitation in the study area by averaging the amount of precipitation in the current and preceding 2 months, which we viewed as a balance between detecting the immediate effects of precipitation to provide drinking water for animals and longer‐term effects on vegetation growth (Olivier et al., 2022).

Surface water availability within the salt pans was assessed by calculating the Normalized Difference Water Index (NDWI) that uses a ratio of green and infrared bands (GREEN − NIR/GREEN + NIR) to delineate open water features (Ji et al., 2009; McFeeters, 1996). Generally, positive NDWI values correspond to surface water and negative values represent non‐aqueous surfaces. The NDWI values were derived using the USGS Landsat 8 satellite imagery (30‐m resolution) at a 16‐day temporal resolution. We replaced any cloud cover in the satellite images with missing data, calculated the NDWI for each pixel in the image set, and created a mosaic of the average pixel value for each month. We then calculated the average monthly NDWI value within the intersection of the salt pan boundaries and the study area to represent the relative amount of water available to wildlife. We assessed surface water availability in the salt pans only because it is difficult to detect surface water using NDWI in vegetated areas, such as the grasslands and woodlands of the study area where wildlife might find water, but we expect that the amount of water in the salt pans is reflective of the surface water in the area more generally.

Finally, we estimated primary productivity and thus forage availability for wild herbivores and livestock, using the Normalized Difference Vegetation Index (NDVI) time series dataset at 8‐day resolution provided by MODIS Land Products data (250‐m resolution). We calculated the average monthly NDVI in two ways: (1) within 10 km of the cattle‐posts (NDVI_cp_) to provide the most comprehensive measure of foraging available to livestock, and (2) across the entire spatial extent of the study area (NDVI_sa_), because wild prey are not tied to cattle‐post locations and some analyses aggregated the PAC reports across cattle‐posts. We tested the sensitivity of our extraction of NDVI_cp_ at three buffer sizes: 3 km, 5 km, and 10 km radii, but found that all three buffer distances provided similar results and chose to use NDVI_cp_ within 10 km of the cattle‐posts to provide the most comprehensive measure of foraging available to livestock.

### 2.6 Statistical analyses

We tested for differences in the average monthly PAC reports per cattle‐post between peak wet (Dec.–Feb.) versus peak dry (Aug.–Oct.) seasons and among years using a Mann–Whitney *U* test and a Kruskal–Wallis test, respectively. Nonparametric tests were used because monthly PAC data were not normally distributed among groups. We also tested for the effects of proximity to the national park boundary (scaled) on the total number of PAC reports per cattle‐post using a generalized linear model.

We used three modeling approaches to assess the effects of resources on wild prey availability and livestock depredation: (1) A binomial logistic regression model to test the effects of primary resource availability on monthly wild prey availability in the study area, using the binomial family with the response variable constructed as the number of “successes” (number of wild prey GPS locations occurring in the study area) and “failures” (number of GPS locations outside of the study area) per month (Douma & Weedon, 2019). (2) A zero‐inflated Poisson mixed effects model, which is most appropriate for count data such as those analyzed here, to test the effects of primary resource availability on the number of monthly PAC reports at each cattle‐post, including cattle‐post ID as a random effect using R package “GLMMadaptive” (Rizopoulos, 2023). We included zero inflation to account for many months with zero PAC reports when separated by cattle‐post (79.9% of observations), and the effects of environmental variables were included for the Poisson component of the model only. We found no evidence of overdispersion in the models, confirming that a Poisson distribution was appropriate over a negative binomial distribution. (3) A Poisson regression model to test the effects of both wild prey and primary resource availability on the total number of PAC reports (aggregating all cattle‐posts) for months with wild prey availability data (*N* = 32). This model did not include zero inflation because there was a low proportion of months with zero PAC reports in the cattle‐post aggregated dataset (28.1% of observations).

In each analysis, we compared all combinations of a global model including the relevant explanatory variables as predictors: average NDVI (across the study area [NDVI_sa_] or surrounding cattle‐posts [NDVI_cp_] depending on the scale of the response variable), average NDWI of the salt pans, lagged precipitation (3‐month average of total precipitation), and the proportion of wild prey locations in the study area. Model 3's global model additionally included interaction terms between wild prey availability and the environmental variables to reflect that the effects of primary resources could depend on wild prey availability and that the two are inherently linked. For example, inundated habitats could make wild prey more difficult to catch for lions, resulting in higher livestock depredation (Olivier et al., 2022), but we expect this hypothetical relationship would have no effect on observed livestock attacks if wild prey were unavailable to begin with.

All environmental variables were scaled and centered (mean = 0, std = 1) to produce comparable model coefficients. Because the lagged precipitation and average NDVI were highly correlated (*r* > |.6|), we excluded models with both variables during model evaluation. We compared candidate models using the Akaike Information Criterion (AIC) or AIC corrected for small sample sizes (AIC_c_) for models with *N* < 50. We considered models to be similar in performance if they did not differ by >2 ΔAIC/AIC, but focused our interpretation on the lowest AIC/AIC_c_ model in each set (Burnham et al., 2011). We evaluated model fit by calculating McFadden's pseudo‐*R* ^2^ for the binomial logistic and Poisson regression models, and visually inspected the zero‐inflated Poisson regression model fit using Q–Q plots (Rizopoulos, 2023). We also tested the sensitivity of our use of lagged precipitation by comparing models using lagged precipitation to those with precipitation for the previous month and precipitation in the current month, but we found that including lagged precipitation produced the best performing models for all model sets.

## 3 RESULTS

**FIGURE 2:**
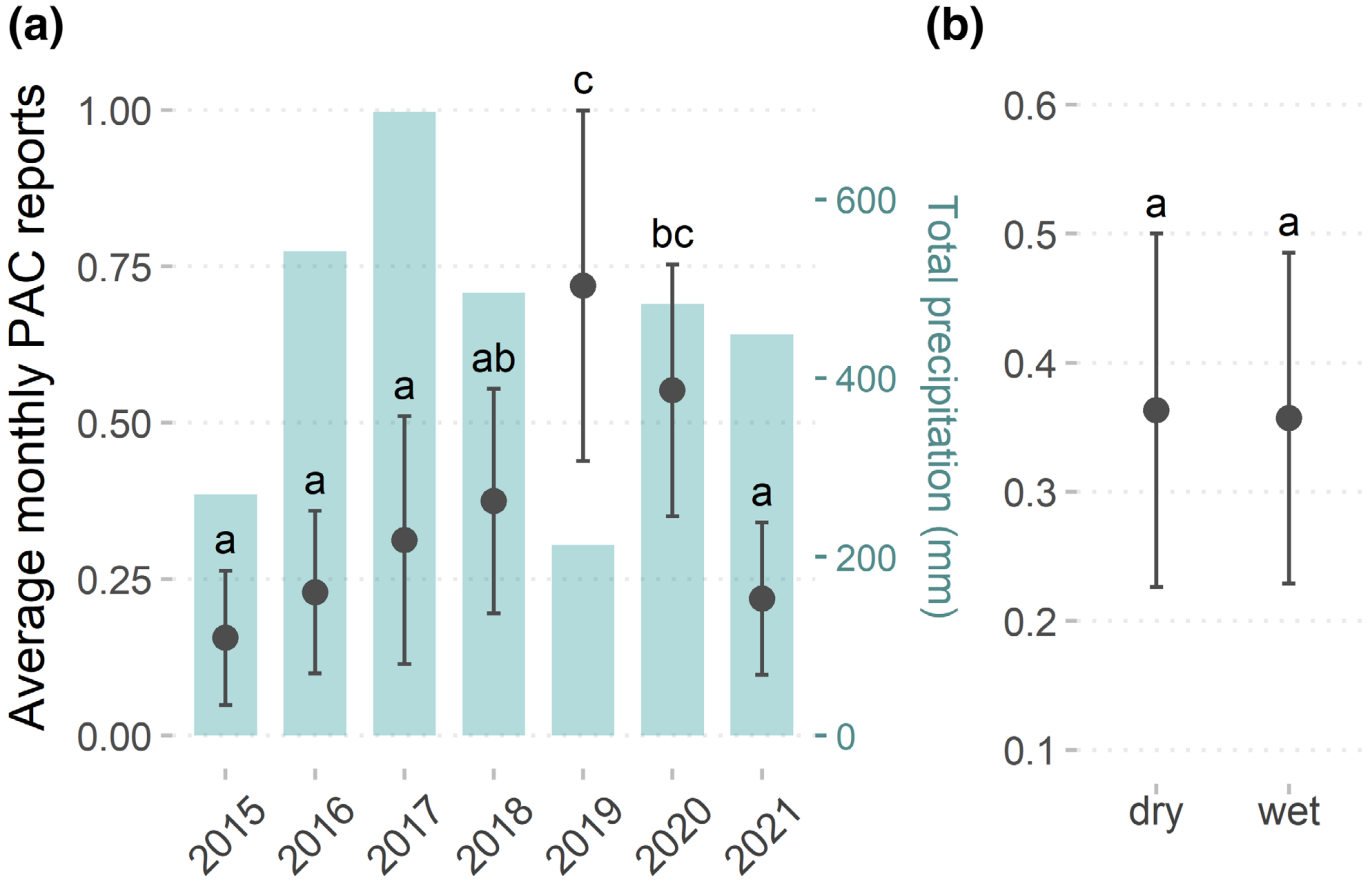
Differences in average monthly livestock depredation reports by African lions per cattle‐post (a) among years and (b) between peak wet versus dry seasons, for eight focal cattle‐posts in the eastern Makgadikgadi Salt Pans ecosystem of Northern Botswana from 2015 to 2021. Total precipitation in the study area per year is represented by blue bars and on the right‐hand axis in (a). Error bars represent 95% CIs. Letters above CIs indicate significant differences among groups according to (a) Kruskal–Wallis tests or (b) Mann–Whitney *U*, respectively.

From 2015 to 2021, a total of 246 livestock depredation reports were attributed to lions at the eight focal cattle‐posts in the study area. We collected questionnaire responses from 26 independent cattle‐post residents (i.e., from separate households) at seven of the eight cattle‐post locations, who reported ownership of a total of 1449 cattle, 480 goats/sheep, and 184 other livestock (i.e., donkeys, horses, etc.) in the study area in 2022. Increases in livestock depredation by predators in the last 10 years were reported by 22 respondents (84.6%), while only two reported decreased depredation and two reported no difference in depredation rates (7.69% each). Monthly reports of depredation by lions per cattle‐post generally increased over time with the highest levels of depredation reported in 2019 and 2020 (Kruskal–Wallis χ ^*2*^ = 31.64, df = 6, *p* < .001), though there was a notable decline in reports in 2021 (Figure 2a). We found no differences in the number of livestock depredation reports between the wet and dry seasons (Mann–Whitney *U* test, *p* = .75; Figure 2b); but 15 cattle‐post residents (57.7%) reported higher depredation rates in the dry season compared to 7 (26.9%) reporting higher rates in the rainy season and 4 (15.4%) reporting no difference between seasons. There was also no effect of the distance to the national park boundaries on the total number of PAC reports at each cattle‐post (*β* = −1.28, 95%CI: −14.06, 11.50; *p* = .85).

**FIGURE 3:**
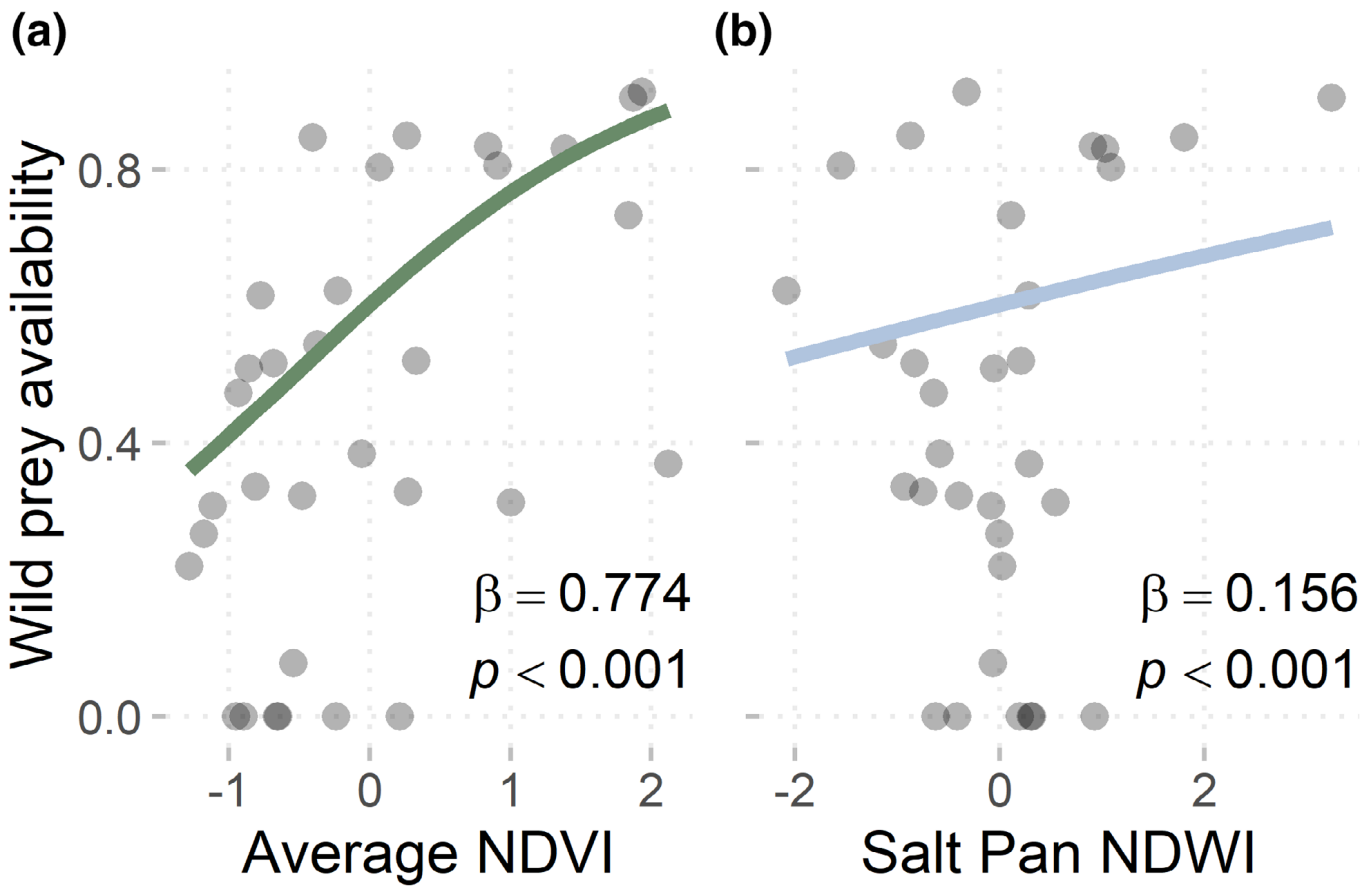
Fitted effects of (a) NDVI and (b) salt pan NDWI in the study area on monthly prey availability (i.e., wildebeest and zebra) in the eastern Makgadikgadi Salt Pans ecosystem of Northern Botswana from 2016 to 2019. Fitted relationships (with very small 95% confidence intervals) and statistics (*β* coefficient and associated *p*‐value) represent the top performing binomial logistic regression model results. Wild prey availability on *y*‐axis is measured as the proportion of wild prey GPS locations occurring in the study area per month, with each point representing 1 month of data for all collared individuals (*N* = 32). On *x*‐axes, Average NDVI is the scaled value of monthly average NDVI within the study area, and Salt pan NDWI is the scaled value of monthly average NDWI within the salt pan and study area boundaries.

The lowest AIC_c_ logistic regression model for wild prey (*R* ^2^ = .48, AIC_c_ weight = 1) showed that relative wild prey availability—or the monthly proportion of wild prey GPS points occurring in the study area—increased as NDVI_sa_ increased (*β* = .774, 95%CI: 0.764, 0.784; *p* < .01; Figure 3a) and as salt pan NDWI increased (*β* = .155, 95%CI: 0.147, 0.164; *p* < .01; Figure 3b, Table 1), with each 0.1 increase in unscaled NDVI_sa_ and salt pan NDWI resulting in a 10% and 4.6% increase in wild prey availability, respectively.

**TABLE 1:** Statistical models within <2 ΔAIC/AIC_c_ of the top‐performing model evaluating the effects of primary resource availability on monthly prey abundance and livestock depredation, measured by the number of PAC reports, including scaled model coefficients (with 95% CIs).

		Model coefficients (95% CI)				Model comparison	
		Average NDVI	Average salt pan NDWI	Lagged precipitation	Prey availability	ΔAIC/AIC_c_	Weight
Prey abundance models		NDVI_sa_					
Binomial logistic	NDVI_sa_ + NDWI	0.774* (0.76, 0.78)	0.156* (0.15, 0.16)	–	–	0	1
Cattle‐post PAC reports models		NDVI_cp_					
Zero‐inflated poisson^a^	NDVI_cp_ + NDWI	−0.140 (−0.34, 0.04)	−0.256* (−0.46, −0.05)	–	–	0	0.38
	NDWI	‐	−0.269* (−0.46, −0.07)	–	–	0.4	0.31
	NDWI + Precip	‐	−0.262* (−0.46, −0.06)	−0.101 (−0.26, 0.06)	–	0.9	0.24
Total PAC reports models		NDVI_sa_					
Poisson	NDWI + Precip + Prey^†^	–	0.009 (−0.28, 0.30)	0.422* (0.18, 0.65)	−0.261* (−0.52, −0.01)	0	0.66
	Prey:NDWI + Prey:Precip	–	Prey: −0.391* (−0.71, −0.08)	Prey: −0.404* (−0.67, −0.17)			

*Note*: Coefficient significance: *p < .05, ^†^ *p* < .1. Average NDVI_sa_: scaled monthly average of NDVI across the study area extent. Average NDVI_cp_: scaled monthly average of NDVI within 10 km of a cattle‐post. Salt pan NDWI: scaled monthly average of NDWI within the salt pan and study area boundaries. Lagged precipitation: scaled 3‐month average of total monthly precipitation. Prey availability: scaled monthly proportion of collared prey GPS locations occurring within the study area.

^a^ Zero‐inflated Poisson models include a random effect of cattle‐post ID.

**FIGURE 4:**
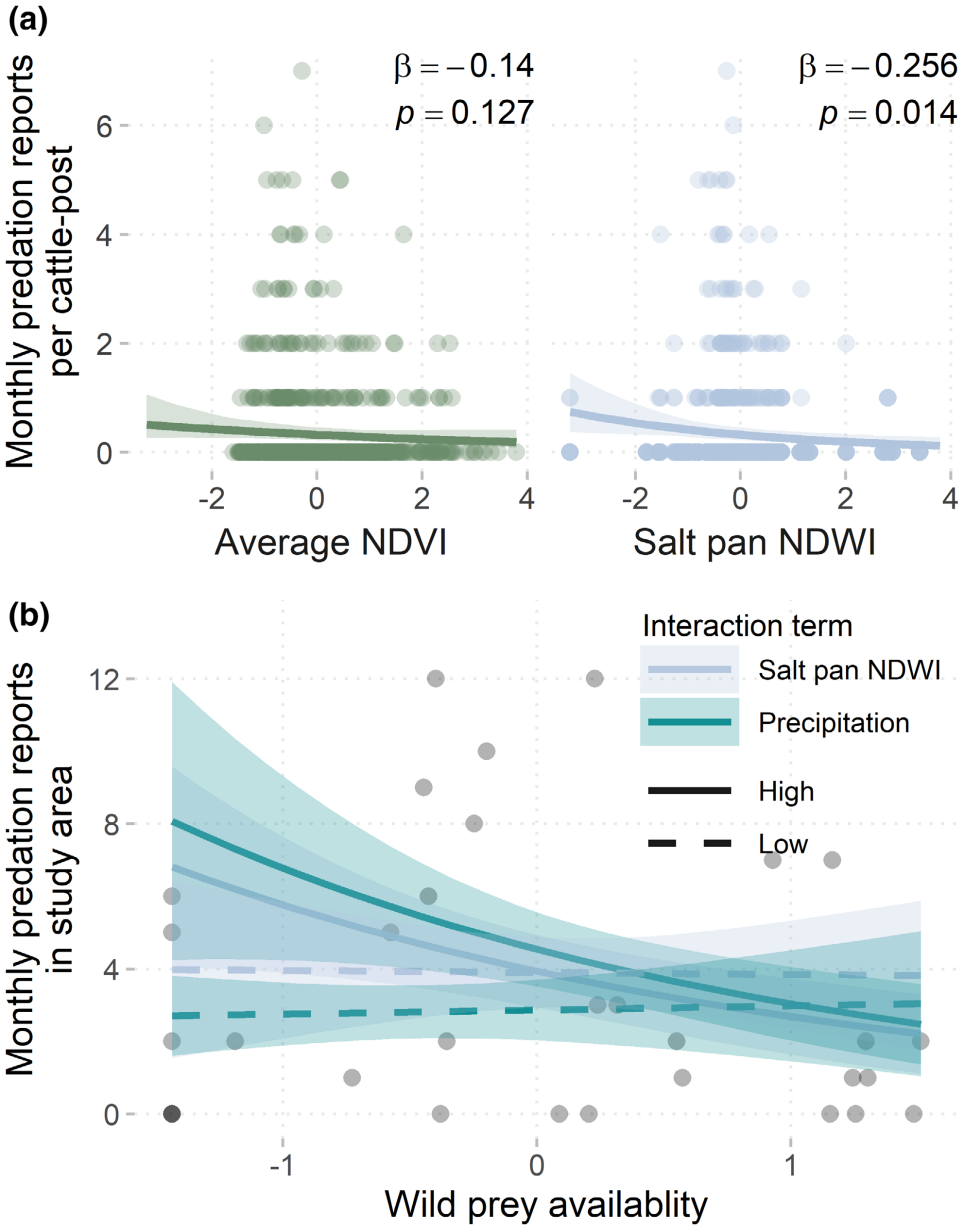
The effects of primary resources and prey availability on the monthly total of livestock depredation reports attributed to lions for eight cattle‐posts in the eastern Makgadikgadi Salt Pans ecosystem of Northern Botswana from 2015 to 2021. (a) Fitted effects (with 95% CIs and model coefficients) of NDVI and salt pan NDWI on the number of PAC reports per cattle‐post from the top performing zero‐inflated Poisson mixed‐effects model, including cattle‐post ID as a random effect. On *x*‐axes, Average NDVI is the scaled value of monthly average NDVI within 10 km of a cattle‐post, and Salt pan NDWI is the scaled value of monthly average NDWI within the salt pan and study area boundaries. (b) Model predictions for the top performing Poisson regression model for the total number of PAC reports in the study area as a function of scaled wild prey availability, under low and high conditions of primary resource availability. Fitted relationships (with 95% CIs) were calculated for significant interaction effects of prey availability with salt pan NDWI and lagged precipitation by generating model predictions for the 25% (low) and 75% (high) quartiles of the environmental predictors.

The number of livestock depredation reports at each cattle‐post was best predicted by the combination of water availability and primary production over the full 7‐year study period (AIC weight = 0.38, Table 1). Zero‐inflated Poisson mixed effects modeling indicated that livestock depredation reports were highest during months with low salt pan NDWI (*β* = −0.256, 95%CI: −0.46, −0.05; *p* = .01) and low average NDVI at cattle‐posts (NDVI_cp_, *β* = −.140, 95%CI: −0.32, 0.04; *p* = .13; Figure 4a), though the latter was not a significant effect. In other words, a 0.1 increase in unscaled salt pan NDWI or NDVI_cp_ reduced the expected count of monthly PAC reports at a given cattle‐post by 7.1% and 1.4%, respectively. We also detected a non‐significant negative effect of lagged precipitation on cattle‐post PAC reports in the top model set (ΔAIC = 0.9, model weight = 0.24, Table 1), suggesting that increasing precipitation might similarly reduce the likelihood of livestock depredation events (*β* = −.101, 95%CI: −0.26, −0.06; *p* = .23).

At the study area scale, the relationship between primary resources and livestock depredation reports interacted with monthly wild prey availability. The top performing Poisson regression model (*R* ^2^ = .21, AIC_c_ weight = 0.66) showed that total PAC reports across the study area were highest at times of low wild prey availability (*β* = −.261, 95%CI: −0.52, −0.01; *p* = .04). However, both salt pan NDWI and lagged precipitation significantly altered the effects of wild prey availability (*β*_Prey:NDWI_ = −0.391, 95%CI: −0.71, −0.08; *p* = .01; *β*_Prey:Precip_ = −.404, 95%CI: −0.67, −0.17; *p* < .01). The reduction in total PAC reports with increasing prey availability was only apparent at times of high water availability, as measured by salt pan NDWI and lagged precipitation (Figure 4b). When lagged precipitation and salt pan water availability was low, however, prey availability had negligible impacts on livestock depredation reports. Additionally, higher levels of precipitation in the preceding 3 months increased the likelihood of livestock depredation events by lions in the study area (*β* = −.422, 95%CI: −0.18, −0.65; *p* < .01). This finding contrasted with results from the model for PAC reports per cattle‐post which suggested a non‐significant but negative effect of lagged precipitation. Finally, the direct effect of salt pan NDWI on total study area PAC reports was negligible (*β* = .009, 95%CI: −0.28, 0.30; *p* = 95), suggesting the importance of considering the interaction between salt pan NDWI and wild prey availability over the direct impact of salt pan water availability.

## 4 DISCUSSION

Our results suggest that primary resource availability in combination with the availability of wild prey were important in shaping rates of livestock depredation by lions in the Makgadikgadi Pans ecosystem. We found that livestock depredation is most likely when water and forage resources are limited and may cause wildlife and livestock to adjust their foraging behaviors. Increased wild prey availability generally served as a buffer to livestock populations that reduced livestock depredation reports, potentially indicating that prey switching by lions is a mechanism driving depredation events. However, the effect of wild prey availability on depredation reports was dependent on the abundance of water resources in the study area, and wild prey availability itself was determined by forage and water resources. Our findings could be explained by foraging ranges and activity of wild prey, livestock, and lions that are fundamentally driven by primary resources, shaping both livestock and wild prey vulnerability to predation. Given the predicted redistribution of primary resources in space and time due to ongoing climate change, particularly increasing drought prevalence and reduced primary productivity, as well as ongoing prey depletion in many African systems, our results echo recent projections of increasing human–carnivore conflict (Abrahms et al., 2023; Creel et al., 2018; Hulme et al., 2001; Wolf & Ripple, 2016; Wu et al., 2021).

Though our results establish links between decreased resource availability, reduced wild prey availability, and increased livestock depredation events (Figures 3 and 4a), we also found that when surface water was limited, wild prey availability did not predict livestock depredation reports (Figure 4b). Like wild prey, cattle distribution and movement can be strongly driven by the availability of forage and water resources, particularly in semi‐arid systems such as the Makgadikgadi Pans (Butt, 2010; Manning et al., 2017; Scoones, 1995; Tyrrell et al., 2017; Weise et al., 2019). However, wild prey and livestock in this study area respond to limited resource availability in different ways. While wild prey can leave to seek out resources elsewhere (such as the other side of MPNP), livestock must regularly return to their home cattle‐posts and likely extend their ranging behaviors in search of foraging opportunities. Although livestock movement data for our study system was unavailable, in‐depth interviews with cattle‐owners to support participatory mapping research on the west side of MPNP suggest that free‐roaming cattle increase their home ranges during the dry season when resources are scarce (K. Orrick, *unpublished data*). Similarly, cattle in Bostwana's Okavango Delta have been observed graze further from settlements as water resources dwindle in the dry season (Weise et al., 2019). Even in systems where livestock are actively herded and protected, livestock space use is driven by selection for foraging resources (Butt, 2010; Feldt & Schlecht, 2016) and so livestock foraging and movement behaviors can still contribute to herd vulnerability to predation, though these relationships are likely buffered by more active defense of the herds by people and guardian animals (Weise et al., 2019; Woodroffe et al., 2007). Most studies investigating the effects of primary resources on livestock depredation do so in the context of resource effects mediated through wild prey availability; the effects of resource availability on livestock ecology are generally overlooked (Olivier et al., 2022; Patterson et al., 2004; Wilkinson et al., 2020). Our findings highlight that changes in livestock space use and foraging activity may be an integral component of understanding livestock depredation rates.

Environmental conditions that shape landscape accessibility and benefit ambush hunting strategies for lions often contribute to livestock vulnerability to predation, highlighted by our finding that higher levels of recent precipitation led to increased monthly livestock depredation events (Beattie et al., 2020; Owen‐Smith, 2015). Recent rainfall can result in enhanced vegetation growth and stalking cover for lions as well as flooding that is common in the Makgadikgadi ecosystem, which could affect the accessibility and relative vulnerability of livestock and wild prey. Notably, our results contrast with the broader trend in which the year with the lowest total rainfall in the study area (2019) corresponded to the year with the highest levels of livestock predation reports (Figure 2a), which further suggests that fine‐scale assessments of temporal trends are important in understanding the drivers of conflict in this system. Migratory wild prey were less likely to migrate away from the study area in recent years due to the installation of artificial waterholes in the area that provide drinking water year‐round, reducing the efficacy of using simple wet versus dry season comparisons (Bennitt et al., 2022). If resident wild prey are less dependent on waterholes following bouts of rain and become more widely dispersed across the landscape, livestock may then be relatively more accessible to lions if they have more predictable and clustered spatial distributions than wild prey.

Because livestock are not actively herded in our study area, they forage without human‐imposed restrictions and effectively serve as resident prey (as opposed to migratory prey) with a unique ecology (e.g., different energetic demands and spatiotemporal activity patterns than wild prey). Unfortunately, data on livestock and lion movement throughout the study area were unavailable, and so we cannot definitively say how livestock and lion foraging behaviors in response to resources directly contributed to livestock depredation risks. It also remains unknown whether livestock depredation in our study system is driven by the behaviors of specific lion individuals, prides, or age/sex classes. But our findings broadly suggest that livestock serve as a prey resource for a generalist predator (i.e., lions) that increases kill rates on livestock not only because wild prey are less available, but because livestock become more accessible and vulnerable to predation. This is in line with Miller and Schmitz's (2019) conceptualization of predator–prey interaction theory in the context of livestock depredation, but our findings extend this theoretical framework to consider depredation driven by habitat domain shifts in livestock in addition to predators and wild prey.

Reductions in resource availability due to climate change are likely to cause more risk‐taking behaviors in lions and other large carnivores, possibly resulting in higher incidences of human–carnivore conflict (Mills et al., 2023; Tuqa et al., 2014). Increased livestock depredation rates may reflect larger lion home ranges and increased overlap with high‐risk, human‐dominated areas, incidentally leading to higher encounter rates with livestock. In many African systems, the depletion of wild prey can be decoupled from primary resource availability due to intense offtake pressures from poaching and subsistence hunting (Craigie et al., 2010; Lindsey et al., 2017), leading to carnivore dietary shifts toward smaller prey items (Creel et al., 2018). Given the overall negative effect of wild prey availability on livestock depredation in our study area (Table 1), we reiterate long‐standing concerns that broad‐scale trends in wild prey depletion will amplify human–carnivore conflict in many systems (Khorozyan et al., 2015; Wolf & Ripple, 2016).

Though resource availabilities clearly underpin livestock depredation rates in Makgadikgadi and elsewhere, a number of other factors can also influence lion depredation of livestock. The growth of cattle herds over time could increase rates of livestock killings, though cattle owners in our study area did not report any increases in their numbers of cattle in recent years. Additionally, the demographic makeup of local lion populations may lead to changes in prey preferences, such as when smaller prides or those with young cubs preferentially seek out easier‐to‐capture prey items, which could exacerbate livestock depredation (Scheel, 1993). Importantly, the combination of lion, livestock, and wild prey responses to limited resource availabilities may be synergistically contributing to escalated livestock depredation. Future work that integrates wildlife and livestock movements in response to environmental heterogeneity at fine spatiotemporal scales would help further tease apart these complex relationships. However, it is important to note that reports of livestock depredation could also fluctuate based on socio‐economic or policy changes (e.g., compensation rates), though we are not aware of any such changes and would not expect those to vary in response to any of our explanatory variables.

Our results run counter to previous work in MPNP and elsewhere that suggests clear seasonal trends (e.g., wet vs. dry season) in livestock depredation rates (Valeix et al., 2012), indicating that environmental heterogeneity shapes conflict at finer temporal scales. Cattle‐post residents also reported generally higher livestock depredation by predators in the dry season months, a trend that was not observed in the PAC data for lion depredation events. As such, seasonal recommendations for conflict mitigation measures are likely to be ineffective relative to strategies tailored to recent and predicted environmental conditions. Although employing herdsmen year‐round or seasonally may not be financially feasible for cattle‐owners, it may be beneficial at lower costs to adaptively herd livestock in months of increased depredation risk when vegetation productivity and water resources have declined (Olivier et al., 2022). Residents around MPNP often look to governmental intervention as a solution to conflict, such as large‐scale fencing to limit wildlife movement between MPNP and livestock grazing areas (Hemson et al., 2009). Our results suggest some areas for future research or for policy makers to consider. Tourism operators and government agencies could tailor common ecosystem management strategies to mitigate conflict in response to dynamic environmental conditions. For example, the provisioning of water resources via boreholes can influence herbivore distributions (Chamaillé‐Jammes et al., 2016; Smit et al., 2007), though this could adversely impact other wildlife as well as fundamental ecological processes that govern the ecosystem (Bennitt et al., 2022; Selebatso et al., 2018). Given that lions generally select for waterholes as hunting grounds (Davidson et al., 2013; Valeix et al., 2010) and largely prey upon livestock opportunistically and prefer wild prey (Beattie et al., 2020; Everatt et al., 2023; Valeix et al., 2012), strategically timing the pumping of existing boreholes in the Makgadikgadi system—particularly those farthest from cattle‐posts—could encourage spatial separation of lions and livestock at times of high conflict risk. However, the use of boreholes can disrupt the natural migratory movement of wildlife that track water resources in this system, and so further studies are necessary to investigate the best practices for using boreholes as a management intervention.

A common assumption is that wild prey availability is the primary driver of livestock depredation by large carnivores (Khorozyan et al., 2015), but our results enhance this narrative by showing that these patterns are likely underpinned by wildlife and livestock responses to fluctuations in primary resources. The co‐occurrence of wildlife and free‐roaming cattle generates complex ecological interactions in which livestock, wild prey, and predators must all adaptively respond to changes in resource availability. As climate change limits resource availabilities worldwide, expanding home ranges and novel movement patterns of wildlife and livestock alike will likely lead to intensified human–carnivore conflict (Abrahms et al., 2023; Tucker et al., 2018). Our results highlight the synergistic threats of climate change amid growing concerns for the conservation of biodiversity and human wellbeing in coming years (Martens et al., 2022; Pecl et al., 2017). However, using insights from ecological theory to better understand human–wildlife conflict, one facet of climate change impacts, can help inform mitigation strategies for a dynamic and uncertain future.

## AUTHOR CONTRIBUTIONS

**Kirby L. Mills:** Conceptualization (lead); data curation (lead); formal analysis (lead); funding acquisition (lead); investigation (lead); methodology (equal); visualization (lead); writing – original draft (lead); writing – review and editing (lead). **Emily Bennitt:** Conceptualization (supporting); data curation (equal); funding acquisition (equal); supervision (equal); writing – review and editing (equal). **Kai Zhu:** Investigation (supporting); methodology (supporting); validation (equal); writing – review and editing (equal). **Hattie L. A. Bartlam‐Brooks:** Data curation (equal); funding acquisition (equal); writing – review and editing (equal). **Tatjana Y. Hubel:** Data curation (equal); funding acquisition (equal); writing – review and editing (equal). **Alan M. Wilson:** Data curation (equal); funding acquisition (equal); writing – review and editing (equal). **Neil H. Carter:** Conceptualization (equal); methodology (equal); supervision (equal); writing – original draft (supporting); writing – review and editing (equal). **Nathan J. Sanders:** Conceptualization (equal); methodology (equal); supervision (equal); writing – original draft (supporting); writing – review and editing (equal).

## FUNDING INFORMATION

Funding for wildlife telemetry study provided by European Research Council Grant 323041. K.L.M. funded by NASA Biodiversity Program (grant no. 80NSSC21K1940). Fieldwork is supported by funding from the American Society of Mammalogists, the University of Michigan African Studies Center, the University of Michigan International Institute, and Rackham Graduate School.

## CONFLICT OF INTEREST STATEMENT

The authors declare no conflicts of interest.

## Supporting information


Data S1.

## Data Availability

Data and code necessary for the analyses in this study are available as Data S1. Conflict and telemetry data will be made available without georeferenced locations and only as post‐processing outputs to safeguard sensitive information related to specific locations of cattle‐posts and conflict.
